# Supplementary material for: Ab Initio Rovibrational Spectroscopy of the Acetylide Anion
Source: Molecules. 2023 Jul 27;28(15):5700. doi: 10.3390/molecules28155700 (PMC10420331; doi:10.3390/molecules28155700)
Supplement: Supplementary file 1 [file molecules-28-05700-s001.zip › molecules-2512527-supplementary.pdf]

Supporting information for:

Ab initio rovibrational spectroscopy of the acetylide anion

Benjamin Schröder*,[a]

[a] *Universität Göttingen, Institut für Physikalische Chemie, Tammannstraße 6, 37077 Göttingen, Germany.*

* E-mail: bschroe4@gwdg.de

Table S1. Mass-independent contributions to the composite potential energy surface (PES) of HCC^-.[a,b]

			$C_{ijk}^{(\alpha)}$					
i	j	k	F12bs	CV	SR	(Q)-(T)	Q-(Q)	P-Q
1	0	0	-0.0012741856	0.0011075260	0.0000788009	-0.0000603928	0.0000503132	-0.0000247543
2	0	0	0.1944060241	-0.0007659326	-0.0001532985	-0.0002348990	0.0000553633	-0.0000166460
3	0	0	-0.1992058088	0.0004876688	0.0000833562	-0.0001613771	0.0000363994	-0.0000046559
4	0	0	0.1335286541	-0.0002373001	-0.0000523308	-0.0000655344	0.0000086112	-0.0000022532
5	0	0	-0.0775113322	0.0000365648	0.0000220637	0.0000047688	-0.0000019398	0.0000031291
6	0	0	0.0415916571	0.0000285932	0.0000077222	0.0000245552	0.0000408205	-0.0000034280
7	0	0	-0.0214796489					
8	0	0	0.0080958599					
0	1	0	-0.0039348455	0.0051918677	0.0002987990	-0.0013032382	0.0003356066	-0.0002728501
0	2	0	0.4123913599	-0.0038900738	-0.0005831154	-0.0009147351	0.0005067056	-0.0003333826
0	3	0	-0.4392756871	0.0020032748	0.0003966994	-0.0004052099	0.0004461332	-0.0002781899
0	4	0	0.2876702492	-0.0008466923	-0.0001946532	-0.0002490661	0.0002145797	-0.0001397859
0	5	0	-0.1526279908	0.0002995503	0.0000727552	-0.0000209999	0.0000474519	0.0000119853
0	6	0	0.0713720049	-0.0000339600	0.0000365622	0.0004097648	0.0000770382	0.0001598423
0	7	0	-0.0286758205					
0	8	0	0.0072523185					
0	0	2	0.0158451783	-0.0000399204	-0.0000310217	-0.0002931333	0.0000061790	-0.0000082950
0	0	4	0.0016589413	0.0000113278	0.0000017318	0.0000246029	0.0000294853	0.0000206180
0	0	6	-0.0006107949	0.0000009989	-0.0000005858	0.0000216849	-0.0000124661	-0.0000044199
0	0	8	-0.0000963453					
0	0	10	0.0000094614					
1	1	0	-0.0120470892	0.0001371425	-0.0000453075	0.0000276216	0.0001364410	-0.0000573050
2	1	0	0.0009113801	-0.0000563114	-0.0000221334	-0.0002433545	0.0001234697	-0.0000446642
1	2	0	-0.0029657570	-0.0000317631	-0.0000076220	0.0000587778	0.0001496450	-0.0000800592
3	1	0	-0.0047931659	-0.0000014513	0.0000133990	-0.0001274547	0.0000568776	
2	2	0	-0.0051410170	0.0000159346	0.0000205121	0.0000114493	0.0001406436	
1	3	0	0.0019056086	0.0000113740	0.0000102126	0.0000492792	0.0000635691	
4	1	0	0.0022605404	-0.0000525013	0.0000014649	-0.0000976811		
3	2	0	0.0047800092	-0.0000041714	0.0000027256	-0.0000823304		
2	3	0	0.0035076991	-0.0000308231	-0.0000052549	0.0002046453		
1	4	0	-0.0014455376	0.0000529731	-0.0000014649	-0.0002413433		
5	1	0	-0.0013762777					
4	2	0	-0.0019127789					
3	3	0	-0.0023648715					
2	4	0	-0.0030399012					
1	5	0	0.0010596692					
1	0	2	-0.0102079140	0.0000313247	-0.0000114030	-0.0001126849	-0.0000249223	-0.0000249228
0	1	2	-0.0359588230	0.0000424635	0.0000186519	-0.0002633019	-0.0000147663	-0.0000223419
2	0	2	0.0002139101	-0.0000492367	0.0000149474	0.0001141961	0.0000178297	
1	1	2	0.0034916847	-0.0000020127	0.0000167212	0.0001041978	-0.0001644017	
0	2	2	0.0146857046	-0.0000421640	0.0000101230	0.0000393876	-0.0000778833	
3	0	2	0.0019597623	0.0001387546	-0.0000136980	-0.0002396419		
2	1	2	0.0003230593	0.0001109953	-0.0000028107	0.0007329838		
1	2	2	0.0024651403	0.0002650975	-0.0000375689	0.0001249237		
0	3	2	-0.0040329905	-0.0002386586	-0.0000008936	0.0000440732		
1	0	4	0.0001085863	0.0000620130	-0.0000000091	-0.0000813065		

To be continued on next page

Table S1. *Continued from previous page*

i	j	k	$C_{ijk}^{(\alpha)}$ F12bs	CV	SR	(Q)-(T)	Q-(Q)	P-Q
0	1	4	0.0088686198	-0.0000052380	-0.0000089898	-0.0000543644		
4	0	2	-0.0019602207					
3	1	2	-0.0004352388					
2	2	2	-0.0042377622					
1	3	2	-0.0006347079					
0	4	2	0.0037217962					
2	0	4	-0.0008778034					
1	1	4	0.0014198551					
0	2	4	-0.0026947857					

[a] PES coefficients are quoted in atomic units; see Eq. (1) of the main manuscript for their definition.

[b] Linear reference geometry: $r_e = 1.0689$ Å and $R_e = 1.2464$ Å.

Table S2: Diagonal Born-Oppenheimer correction contributions to the composite potential energy surfaces (PESs) of HCC^- isotopologues.[a,b]

			$C_{ijk}^{(\alpha)}$			$C_{ijk}^{(\alpha)}$		
i	j	k	$H^{12}C^{12}C^-$	$H^{13}C^{12}C^-$	$H^{12}C^{13}C^-$	$D^{12}C^{12}C^-$	$D^{13}C^{12}C^-$	$D^{12}C^{13}C^-$
1	0	0	-0.0000778664	-0.0000772205	-0.000077898	-0.0000429279	-0.0000422820	-0.0000429600
2	0	0	0.0001081092	0.0001072019	0.000108076	0.0000601746	0.0000592687	0.0000601419
3	0	0	-0.0000459030	-0.0000453742	-0.000045953	-0.0000260563	-0.0000255295	-0.0000261012
4	0	0	0.0000084526	0.0000080950	0.000008545	0.0000058281	0.0000054573	0.0000059239
5	0	0	-0.0000179318	-0.0000178468	-0.000017912	-0.0000097707	-0.0000096636	-0.0000097705
6	0	0	0.0000395306	0.0000396430	0.000039352	0.0000208245	0.0000209482	0.0000206280
0	1	0	-0.0000169936	-0.0000152255	-0.000015976	-0.0000265366	-0.0000247678	-0.0000255196
0	2	0	0.0000819622	0.0000796322	0.000079607	0.0000713386	0.0000690073	0.0000689848
0	3	0	-0.0000368114	-0.0000355942	-0.000035366	-0.0000356411	-0.0000344435	-0.0000341872
0	4	0	-0.0000067055	-0.0000074752	-0.000007201	0.0000047554	0.0000040273	0.0000042370
0	5	0	-0.0000309608	-0.0000305006	-0.000030334	-0.0000224920	-0.0000218663	-0.0000219538
0	6	0	0.0001532663	0.0001531306	0.000151970	0.0000859164	0.0000853938	0.0000848905
0	0	2	0.0000144973	0.0000137649	0.000013832	0.0000163009	0.0000155683	0.0000156361
0	0	4	0.0000027394	0.0000028742	0.000002820	-0.0000000290	0.0000001061	0.0000000521
0	0	6	-0.0000040663	-0.0000041004	-0.000004086	-0.0000016815	-0.0000017159	-0.0000017020
0	0	8	0.0000011715	0.0000011813	0.000001178	0.0000004783	0.0000004882	0.0000004853
1	1	0	0.0000078856	0.0000076056	0.000007728	0.0000066983	0.0000064070	0.0000065415
2	1	0	0.0000335772	0.0000337578	0.000033690	0.0000311837	0.0000191031	0.0000188773
1	2	0	-0.0000005645	-0.0000003274	-0.000000033	0.0000022016	0.0000015016	0.0000017274

[a] PES coefficients are quoted in atomic units; see Eq. (1) of the main manuscript for their definition.

[b] Linear reference geometry: $r_e = 1.0689$ Å and $R_e = 1.2464$ Å.

Table S3. Adiabatic composite potential energy surfaces (PESs) of HCC^- isotopologues.[a,b]

			C_{ijk}			C_{ijk}		
i	j	k	$H^{12}C^{12}C^-$	$H^{13}C^{12}C^-$	$H^{12}C^{13}C^-$	$D^{12}C^{12}C^-$	$D^{13}C^{12}C^-$	$D^{12}C^{13}C^-$
2	0	0	0.19309573	0.19309586	0.19309567	0.19309573	0.19309586	0.19309567
3	0	0	-0.19853818	-0.19853856	-0.19853820	-0.19853818	-0.19853856	-0.19853820
4	0	0	0.13299097	0.13299128	0.13299104	0.13299097	0.13299128	0.13299104
5	0	0	-0.07733713	-0.07733747	-0.07733709	-0.07733713	-0.07733747	-0.07733709
6	0	0	0.04165314	0.04165351	0.04165295	0.04165314	0.04165351	0.04165295
7	0	0	-0.02144675	-0.02144686	-0.02144675	-0.02144675	-0.02144686	-0.02144675
8	0	0	0.00809586	0.00809586	0.00809586	0.00809586	0.00809586	0.00809586
0	2	0	0.40772789	0.40772845	0.40772717	0.40772789	0.40772845	0.40772717
0	3	0	-0.43755994	-0.43756124	-0.43755992	-0.43755994	-0.43756124	-0.43755992
0	4	0	0.28672029	0.28672119	0.28672074	0.28672029	0.28672119	0.28672074
0	5	0	-0.15240308	-0.15240357	-0.15240299	-0.15240308	-0.15240357	-0.15240299
0	6	0	0.07224655	0.07224685	0.07224550	0.07224655	0.07224685	0.07224550
0	7	0	-0.02869663	-0.02869676	-0.02869670	-0.02869663	-0.02869676	-0.02869670
0	8	0	0.00725232	0.00725232	0.00725232	0.00725232	0.00725232	0.00725232
0	0	2	0.01550121	0.01550058	0.01550059	0.01550121	0.01550058	0.01550059
0	0	4	0.00174633	0.00174645	0.00174640	0.00174633	0.00174645	0.00174640
0	0	6	-0.00060965	-0.00060968	-0.00060967	-0.00060965	-0.00060968	-0.00060967
0	0	8	-0.00009517	-0.00009516	-0.00009517	-0.00009517	-0.00009516	-0.00009517
0	0	10	0.00000946	0.00000946	0.00000946	0.00000946	0.00000946	0.00000946
1	1	0	-0.01183783	-0.01183810	-0.01183798	-0.01183783	-0.01183810	-0.01183798
2	1	0	0.00069812	0.00069835	0.00069825	0.00069812	0.00069835	0.00069825
1	2	0	-0.00288457	-0.00288433	-0.00288405	-0.00288457	-0.00288433	-0.00288405
3	1	0	-0.00485088	-0.00485091	-0.00485089	-0.00485088	-0.00485091	-0.00485089
2	2	0	-0.00494928	-0.00494933	-0.00494929	-0.00494928	-0.00494933	-0.00494929
1	3	0	0.00204613	0.00204613	0.00204614	0.00204613	0.00204613	0.00204614
4	1	0	0.00210970	0.00210972	0.00210970	0.00210970	0.00210972	0.00210970
3	2	0	0.00469489	0.00469492	0.00469490	0.00469489	0.00469492	0.00469490
2	3	0	0.00367702	0.00367706	0.00367704	0.00367702	0.00367706	0.00367704
1	4	0	-0.00164036	-0.00164036	-0.00164037	-0.00164036	-0.00164036	-0.00164037
5	1	0	-0.00137628	-0.00137628	-0.00137628	-0.00137628	-0.00137628	-0.00137628
4	2	0	-0.00191278	-0.00191278	-0.00191278	-0.00191278	-0.00191278	-0.00191278
3	3	0	-0.00236487	-0.00236487	-0.00236487	-0.00236487	-0.00236487	-0.00236487
2	4	0	-0.00303990	-0.00303990	-0.00303990	-0.00303990	-0.00303990	-0.00303990
1	5	0	0.00105967	0.00105967	0.00105967	0.00105967	0.00105967	0.00105967
1	0	2	-0.01035144	-0.01035145	-0.01035144	-0.01035144	-0.01035145	-0.01035144
0	1	2	-0.03620685	-0.03620692	-0.03620689	-0.03620685	-0.03620692	-0.03620689
2	0	2	0.00031404	0.00031403	0.00031404	0.00031404	0.00031403	0.00031404
1	1	2	0.00344535	0.00344534	0.00344535	0.00344535	0.00344534	0.00344535
0	2	2	0.01462115	0.01462117	0.01462117	0.01462115	0.01462117	0.01462117
3	0	2	0.00184135	0.00184137	0.00184135	0.00184135	0.00184137	0.00184135
2	1	2	0.00116660	0.00116663	0.00116662	0.00116660	0.00116663	0.00116662
1	2	2	0.00281397	0.00281399	0.00281397	0.00281397	0.00281399	0.00281397
0	3	2	-0.00423413	-0.00423416	-0.00423415	-0.00423413	-0.00423416	-0.00423415
1	0	4	0.00008788	0.00008788	0.00008788	0.00008788	0.00008788	0.00008788
0	1	4	0.00880268	0.00880269	0.00880269	0.00880268	0.00880269	0.00880269
4	0	2	-0.00196022	-0.00196022	-0.00196022	-0.00196022	-0.00196022	-0.00196022

To be continued on next page

Table S3. *Continued from previous page*

i	j	k	C_{ijk} $H^{12}C^{12}C^-$	$H^{13}C^{12}C^-$	$H^{12}C^{13}C^-$	C_{ijk} $D^{12}C^{12}C^-$	$D^{13}C^{12}C^-$	$D^{12}C^{13}C^-$
3	1	2	-0.00043524	-0.00043524	-0.00043524	-0.00043524	-0.00043524	-0.00043524
2	2	2	-0.00423776	-0.00423776	-0.00423776	-0.00423776	-0.00423776	-0.00423776
1	3	2	-0.00063471	-0.00063471	-0.00063471	-0.00063471	-0.00063471	-0.00063471
0	4	2	0.00372180	0.00372180	0.00372180	0.00372180	0.00372180	0.00372180
2	0	4	-0.00087780	-0.00087780	-0.00087780	-0.00087780	-0.00087780	-0.00087780
1	1	4	0.00141986	0.00141986	0.00141986	0.00141986	0.00141986	0.00141986
0	2	4	-0.00269479	-0.00269479	-0.00269479	-0.00269479	-0.00269479	-0.00269479

[a] PES coefficients are quoted in atomic units; see Eq. (1) of the main manuscript for their definition.

[b] Equilibrium bond lengths ($H^{12}C^{12}C^-$, $H^{12}C^{13}C^-$ & $H^{12}C^{13}C^-$): $r_e = 1.06917$ Å and $R_e = 1.24621$ Å.

[c] Equilibrium bond lengths ($D^{12}C^{12}C^-$ & $D^{12}C^{13}C^-$): $r_e = 1.06912$ Å and $R_e = 1.24621$ Å.

[d] Equilibrium bond lengths ($D^{13}C^{12}C^-$): $r_e = 1.06912$ Å and $R_e = 1.24622$ Å.

Table S4. Contributions to the composite electric dipole moment surface (EDMS) of HCC^-.[a,b]

i	j	k	$D^{(\alpha)}_{ijk}$ F12bs	CV	SR	(Q)-(T)	Q-(Q)	P-Q
0	0	0	-1.2666719364	-0.0023103351	0.0017066814	0.0006571292	-0.0000249026	-0.0003923472
1	0	0	0.1188681948	-0.0034669652	0.0009583566	0.0085122099	-0.0016722105	0.0005032511
2	0	0	0.1642677489	-0.0012814159	0.0006380945	0.0087619372	-0.0021840554	0.0002935772
3	0	0	0.0736117350	-0.0002078867	0.0002986543	0.0074985617	-0.0021207665	0.0002092108
4	0	0	0.0265553408	0.0002887205	-0.0000667098	0.0043390896	-0.0012309699	0.0001847997
5	0	0	-0.0137792263					
0	1	0	0.6564404974	-0.0038235664	0.0008574532	-0.0020731781	0.0010375798	-0.0013934357
0	2	0	-0.0749108830	0.0011436357	-0.0002815927	-0.0079825096	0.0027774982	-0.0020813449
0	3	0	-0.0486190159	0.0005526909	-0.0001557555	-0.0096687541	0.0049298408	-0.0001487644
0	4	0	-0.0180231553	0.0007278599	0.0000935468	-0.0077214940	0.0067365190	0.0031066837
0	5	0	0.0407193949					
0	0	2	0.4211351568	0.0010572420	-0.0004672743	-0.0009595058	0.0009692074	-0.0005847071
0	0	4	-0.0358461102	-0.0002403631	0.0001093035	0.0009198420	-0.0006174766	-0.0000054183
0	0	6	0.0208853878	-0.0000448890	-0.0000301469	-0.0005902663	0.0002291370	0.0000940135
0	0	8	-0.0048946711					
1	1	0	0.0919359111	0.0006227829	0.0002311914	0.0051247419	-0.0034428742	0.0020162037
2	1	0	0.0982227991	0.0012105268	0.0000828023	0.0058980687	-0.0026443060	0.0023945726
1	2	0	0.0200681319	0.0003749510	-0.0004769626	-0.0049910834	-0.0023544974	0.0029626179
3	1	0	0.0458344554					
2	2	0	-0.0453842325					
1	3	0	-0.0504720513					
1	0	2	-0.0162599711	0.0011458085	-0.0004043425	-0.0043168604	0.0006851309	
0	1	2	-0.1245380268	0.0019583496	-0.0004155473	-0.0059122296	0.0036643226	
2	0	2	-0.0690861780					
1	1	2	0.0186583875					
0	2	2	-0.1376856389					
0	0	1	0.5401994927	-0.0025019950	0.0009105385	0.0007200125	0.0005983265	-0.0003820219
0	0	3	-0.1600612771	0.0014768106	-0.0004735567	-0.0025216892	-0.0004553830	-0.0003262692

To be continued on next page

Table S4. *Continued from previous page*

i	j	k	$D_{ijk}^{(\alpha)}$ F12bs	CV	SR	(Q)-(T)	Q-(Q)	P-Q
0	0	5	0.0228874473	-0.0001684685	0.0000872438	0.0005176204	0.0006037382	0.0002815693
0	0	7	-0.0021897420	0.0000123242	-0.0000179535	-0.0000213407	-0.0001767812	-0.0000444623
0	0	9	-0.0003426582					
1	0	1	-0.0435351427	0.0009663774	-0.0001617046	-0.0011434824	0.0005603900	
0	1	1	0.4088264666	-0.0010279791	-0.0005274650	0.0011011312	0.0015724377	
2	0	1	-0.0327089764	0.0015070676	0.0001942939	0.0015893462	0.0036302527	
1	1	1	0.0216112665	0.0005924101	-0.0005924101	-0.0013822902	0.0021721703	
0	2	1	-0.3091624803	0.0024647461	-0.0002047388	-0.0015231089	0.0058682464	
3	0	1	-0.0379730134					
2	1	1	-0.0012419084					
1	2	1	-0.1286097464					
0	3	1	0.0381947414					
1	0	3	-0.0104795085					
0	1	3	-0.2583329522					

[a] EDMS coefficients are quoted in atomic units; see Eq. (2) of the main manuscript for their definition.
[b] Linear reference geometry: $r_e = 1.06917$ Å and $R_e = 1.24621$ Å.

Table S5. Composite electric dipole moment surface (EDMS) of HCC^-.[a,b]

$\mu_\parallel$ i	j	k	$D_{ijk}^{\parallel}$	$\mu_\perp$ i	j	k	$D_{ijk}^{\perp}$
0	0	0	-1.26703571	0	0	1	0.53954435
1	0	0	0.12370284	0	0	3	-0.16236136
2	0	0	0.17049589	0	0	5	0.02420915
3	0	0	0.07928951	0	0	7	-0.00243796
4	0	0	0.03007027	0	0	9	-0.00034266
5	0	0	-0.01377923	1	0	1	-0.04331356
0	1	0	0.65104535	0	1	1	0.40994459
0	2	0	-0.08133520	2	0	1	-0.02578802
0	3	0	-0.05310976	1	1	1	0.02240115
0	4	0	-0.01508004	0	2	1	-0.30255734
0	5	0	0.04071939	3	0	1	-0.03797301
0	0	2	0.42115012	2	1	1	-0.00124191
0	0	4	-0.03568022	1	2	1	-0.12860975
0	0	6	0.02054324	0	3	1	0.03819474
0	0	8	-0.00489467	1	0	3	-0.01047951
1	1	0	0.09648796	0	1	3	-0.25833295
2	1	0	0.10516446				
1	2	0	0.01558316				
3	1	0	0.04583446				
2	2	0	-0.04538423				
1	3	0	-0.05047205				
1	0	2	-0.01915023				
0	1	2	-0.12524313				

To be continued on next page

Table S5. *Continued from previous page*

$\mu_\parallel$				$\mu_\perp$			
i	j	k	$D^{\parallel}_{ijk}$	i	j	k	$D^{\perp}_{ijk}$
2	0	2	-0.06908618				
1	1	2	0.01865839				
0	2	2	-0.13768564				

[a] EDMS coefficients are quoted in atomic units; see Eq. (2) of the main manuscript for their definition.
[b] Linear reference geometry: r_e = 1.06917 Å and R_e = 1.24621 Å.

Table S6. Vibrational term energies (in cm^{-1}) and rotational spectroscopic parameters (in MHz) for selected vibrational states in $\mathrm{H}^{12}\mathrm{C}^{12}\mathrm{C}^-$ obtained from variational calculations.

State	G_v	B_v	$10^3 D_v$	$10^6 H_v$
$(0,2^0,0)$	1011.4	41931.8	103.994	0.185
$(0,2^2,0)$	1028.6	41890.8	101.941	0.185
$(0,3^1,0)$	1520.4	42077.9	107.934	0.206
$(0,3^3,0)$	1555.6	41990.4	102.656	0.206
$(0,1^1,1)$	2309.4	41461.2	99.996	0.152
$(0,2^0,1)$	2806.2	41616.4	104.151	0.181
$(0,2^2,1)$	2823.2	41890.8	102.085	0.181
$(0,0^0,2)$	3590.0	41021.1	97.284	0.113
$(1,1^1,0)$	3699.0	41472.8	99.344	0.167
$(1,2^0,0)$	4180.3	41649.4	103.834	0.201
$(1,2^2,0)$	4196.6	41607.7	101.629	0.201
$(1,0^0,1)$	5001.9	41034.6	96.455	0.119
$(1,1^1,1)$	5486.7	41172.1	99.589	0.161
State		q_v	$10^3 q_v^J$	$10^6 q_v^{JJ}$
$(0,2^\ell,0)$		261.6	-4.962	0.089
$(0,3^\ell,0)$		266.8	-6.328	0.292
$(0,1^1,1)$		257.3	-4.729	0.101
$(0,2^\ell,1)$		260.3	-4.845	0.089
$(1,1^1,0)$		264.5	-5.075	0.116
$(1,2^\ell,0)$		267.8	-5.205	0.101
$(1,1^1,1)$		263.4	-5.078	0.116

Table S7. Total internal partition functions of HCC^- isotopologues at different temperatures T (in K).

	$Q_{tot}(T)$			$Q_{tot}(T)$		
T	$H^{12}C^{12}C^-$	$H^{13}C^{12}C^-$	$H^{12}C^{13}C^-$	$D^{12}C^{12}C^-$	$D^{13}C^{12}C^-$	$D^{12}C^{13}C^-$
1	1.055	1.061	1.064	1.110	1.116	1.124
3	1.886	1.921	1.941	2.190	2.218	2.256
5	2.864	2.925	2.958	3.382	3.430	3.495
10	5.351	5.474	5.541	6.395	6.492	6.622
25	12.849	13.157	13.325	15.465	15.708	16.032
50	25.358	25.975	26.311	30.592	31.078	31.726
75	37.874	38.800	39.304	45.759	46.493	47.463
100	50.449	51.689	52.359	61.209	62.216	63.497
125	63.255	64.824	65.655	77.428	78.760	80.337
150	76.557	78.484	79.471	94.949	96.676	98.538
175	90.641	92.964	94.101	114.221	116.428	118.567
200	105.759	108.527	109.809	135.600	138.380	140.795
225	122.123	125.389	126.815	159.375	162.824	165.521
250	139.908	143.732	145.303	185.788	190.012	192.998
275	159.265	163.710	165.428	215.057	220.168	223.453
300	180.326	185.461	187.328	247.386	253.504	257.101
325	203.214	209.113	211.134	282.976	290.227	294.153
350	228.047	234.786	236.968	322.027	330.548	334.819
375	254.938	262.603	264.950	364.745	374.679	379.315
400	284.004	292.683	295.204	411.339	422.844	427.866
425	315.361	325.149	327.851	462.032	475.272	480.704
450	349.129	360.127	363.019	517.051	532.204	538.072
475	385.430	397.747	400.839	576.638	593.893	600.224

Table S8. Integrated intensities[1] (in $cm^{-2}atm^{-1}$) at $T = 300$ K and vibrational term energy differences ΔG_v (in cm $^{-1}$) for various rovibrational bands in $H^{12}C^{12}C^-$.

Band	Sym.[b]	ΔG_v	A_v	A_v^{PR}	A_v^{Q}
$(0,2^2,0) \rightarrow (0,3^1,0)$	e	491.8	1.369	0.533	0.836
$(0,2^2,0) \rightarrow (0,3^1,0)$	f		1.440	0.880	0.560
$(0,1^1,0) \rightarrow (0,2^0,0)$	e	501.3	23.864	23.864	
$(0,0^0,1) \rightarrow (0,1^1,1)$	e	504.9	0.091	0.046	0.045
$(0,2^0,0) \rightarrow (0,3^1,0)$	e	509.0	6.837	3.792	3.045
$(0,0^0,0) \rightarrow (0,1^1,0)$	e	510.1	514.659	260.794	253.865
$(0,1^1,0) \rightarrow (0,2^2,0)$	e	518.5	39.271	19.043	20.228
$(0,1^1,0) \rightarrow (0,2^2,0)$	f		47.629	23.413	24.216
$(0,2^2,0) \rightarrow (0,3^3,0)$	e	527.0	5.329	2.931	2.398
$(0,2^2,0) \rightarrow (0,3^3,0)$	f		5.274	2.902	2.372
$(0,2^0,0) \rightarrow (0,3^3,0)$	e	544.2	0.029	0.016	0.013
$(0,0^0,1) \rightarrow (0,2^0,1)$	e	1001.7	0.009	0.009	
$(0,1^1,0) \rightarrow (0,3^1,0)$	e	1010.3	9.046	8.779	0.267
$(0,1^1,0) \rightarrow (0,3^1,0)$	f		8.959	8.657	0.302
$(0,0^0,0) \rightarrow (0,2^0,0)$	e	1011.4	53.750	53.750	
$(0,0^0,0) \rightarrow (0,2^2,0)$	e	1028.6	1.081	1.056	0.025

To be continued on next page

Table S8. *Continued from previous page*

Band	Sym.[2]	ΔG_v	A_v	A_v^{PR}	A_v^{Q}
$(0,1^1,0) \to (0,3^3,0)$	e	1045.5	0.032	0.028	0.004
$(0,1^1,0) \to (0,3^3,0)$	f		0.057	0.052	0.005
$(0,0^0,0) \to (0,3^1,0)$	e	1520.4	1.750	0.882	0.868
$(0,0^0,0) \to (0,3^3,0)$	e	1555.6	0.008	0.003	0.005
$(0,0^0,1) \to (0,0^0,2)$	e	1785.5	0.082	0.082	
$(0,2^2,0) \to (0,2^2,1)$	e	1794.6	1.709	1.566	0.143
$(0,2^2,0) \to (0,2^2,1)$	f		1.711	1.568	0.143
$(0,2^0,0) \to (0,2^0,1)$	e	1794.8	1.855	1.855	
$(0,1^1,0) \to (0,1^1,1)$	e	1799.3	20.639	20.015	0.624
$(0,1^1,0) \to (0,1^1,1)$	f		20.513	19.887	0.626
$(0,0^0,0) \to (0,0^0,1)$	e	1804.5	237.684	237.684	
$(0,1^1,0) \to (0,2^0,1)$	e	2296.1	0.177	0.177	
$(0,0^0,0) \to (0,1^1,1)$	e	2309.4	4.606	2.317	2.289
$(0,1^1,0) \to (0,2^2,1)$	e	2313.1	0.302	0.146	0.158
$(0,1^1,0) \to (0,2^2,1)$	f		0.366	0.177	0.189
$(0,0^0,0) \to (0,2^0,1)$	e	2806.2	0.202	0.202	
$(0,0^0,0) \to (0,2^2,1)$	e	2823.2	0.005	0.005	
$(0,2^2,0) \to (1,2^2,0)$	e	3151.7	0.206	0.189	0.017
$(0,2^2,0) \to (1,2^2,0)$	f		0.207	0.190	0.017
$(0,2^0,0) \to (1,2^0,0)$	e	3168.9	0.222	0.222	
$(0,1^1,0) \to (1,1^1,0)$	e		2.146	2.082	0.064
$(0,1^1,0) \to (1,1^1,0)$	f	3188.9	2.132	2.068	0.064
$(0,0^0,1) \to (1,0^0,1)$	e	3197.4	0.004	0.004	
$(0,0^0,0) \to (1,0^0,0)$	e	3209.6	20.820	20.820	
$(0,1^1,0) \to (1,2^0,0)$	e	3670.2	0.198	0.199	
$(0,1^1,0) \to (1,2^2,0)$	e	3686.5	0.306	0.149	0.157
$(0,1^1,0) \to (1,2^2,0)$	f		0.380	0.188	0.192
$(0,0^0,0) \to (1,1^1,0)$	e	3699.0	3.971	2.034	1.937
$(0,0^0,0) \to (0,0^0,2)$	e	3590.0	1.150	1.150	
$(0,0^0,0) \to (1,2^0,0)$	e	4180.3	0.133	0.133	
$(0,0^0,0) \to (1,2^2,0)$	e	4196.6	0.003	0.003	
$(0,1^1,0) \to (1,1^1,1)$	e	4976.6	0.096	0.093	0.003
$(0,1^1,0) \to (1,1^1,1)$	f		0.095	0.092	0.003
$(0,0^0,0) \to (1,0^0,1)$	e	5001.9	1.182	1.182	
$(0,0^0,0) \to (1,1^1,1)$	e	5486.7	0.014	0.007	0.007

[a] Obtained by summation of individual line intensities within a given band and superscripts indicate summations for certain branches omly.

[b] Symmetry/parity label of the lower vibrational state.
